# ADHD symptom trajectories across childhood and early adolescence and risk for hypomanic symptoms in young adulthood

**DOI:** 10.1192/j.eurpsy.2025.24

**Published:** 2025-02-19

**Authors:** Buse Beril Durdurak, Isabel Morales-Muñoz, Georgina Mayling Hosang, Steven Marwaha

**Affiliations:** 1 Institute for Mental Health, University of Birmingham, Edgbaston, Birmingham, UK; 2 Centre for Psychiatry & Mental Health, Wolfson Institute of Population Health, Barts & The London Faculty of Medicine & Dentistry Queen Mary University of London, London UK; 3 Specialist Mood Disorders Clinic, The Barberry Centre for Mental Health, Birmingham and Solihull NHS Trust, Birmingham, UK

**Keywords:** ADHD, ALSPAC, hypomania, LCGA, trajectories

## Abstract

**Background:**

There is increasing evidence that childhood Attention-Deficit Hyperactivity Disorder (ADHD) elevates the risk of later Bipolar Spectrum Disorder (BD). However, it remains unclear whether different trajectories of ADHD symptoms confer differential risk for BD.

**Methods:**

Data from the Avon Longitudinal Study of Parents and Children were available from 7811 children at age 8 years, 7435 at 10, 6798 at 13, and 1217 at 21–23 years. ADHD symptoms were assessed at 8, 10, and 13 years with the Development and Well-Being Assessment. Clinically significant hypomanic symptoms (CSHS) at 21–23 years were assessed using the Hypomania Symptom Checklist (HCL-32). Group trajectories of ADHD and its subtypes were estimated using latent class growth analysis. The prospective associations between different ADHD trajectories and CSHS were tested using logistic regression analysis.

**Results:**

Persistently high, increasing, remitting, and persistently low ADHD symptom trajectories were identified for the three ADHD-related categories. Individuals with persistently high and increasing levels of ADHD symptoms had increased odds of CSHS compared to persistently low class. Sensitivity analyses validated these results. In separate analyses, persistently high levels of hyperactivity and inattentive, and increasing levels of inattentive symptoms were also independently associated with CSHS.

**Conclusions:**

Young people with a longitudinal pattern of high and increasing ADHD symptoms are at higher risk for developing CSHS in young adulthood compared to individuals with low symptom patterns. These two trajectories in childhood and adolescence may represent distinct phenotypic risk profiles for subsequently developing BD and be clinically significant targets for prevention and treatment of BD.

## Introduction

There is growing evidence suggesting that bipolar disorder (BD) is preceded by childhood ADHD [1, 2]. Mania shares many overlapping symptoms with ADHD, such as irritability, increased activity, aggression, problems in social situations, disinhibition, and/or distractibility [3]. It is estimated that up to 1 in 13 patients with ADHD have comorbid BD, up to 1 in 6 patients with BD have comorbid ADHD in adult populations [4] and that about 10% of children and adolescents with ADHD will develop BD [5]. Further, young people with comorbid ADHD and hypomania, or BD, have an increased risk of suicide [6], more psychiatric hospitalizations, less treatment adherence, higher rates of additional psychopathology [5, 7], and an earlier age of onset [4] than individuals without such comorbidity. A recent study also found that most offspring of BD parents did not develop BD, but those with preschool ADHD were at particularly high risk for developing BD [8].

Most studies investigating the association between ADHD and BD development have been limited by measuring ADHD either at one-time point or by simply reporting the diagnostic proportions for the sample at various follow-up times. This method does not capture intraindividual variability in ADHD symptoms or their longitudinal course and could mask a potentially complex association or mechanism. The presence of subgroups may also explain apparent divergent results within the literature. [9–11] This is also important as ADHD is a neurodevelopmental disorder starting early in life and developing with a highly variable trajectory. [12–16] It remains unclear whether different trajectories of ADHD symptoms confer differential risk for development of BD. Further, although modest correlations between adolescent hypomanic and hyperactivity symptoms have been reported, recent research has detected higher estimates for genetic risk factors between hypomania and symptoms of hyperactivity (10–25%) than with inattention (6–16%). [1] Another study found that BD was associated with inattentive and combined but not with hyperactive ADHD presentations [17]. Thus, since the ADHD symptom domains of hyperactivity and inattention may be differentially associated with BD and there are incongruent findings in the literature, ideally these domains need to be further investigated separately.

Understanding unique trajectories of ADHD symptoms and how these subgroups influence the development of BD could help identify at-risk groups and could guide specific interventions. One way to identify the earliest clinical manifestations of BD is to study hypomanic symptoms, a common feature of BD in youth which often heralds a subsequent manic episode [18] Recent research has found that traits of ADHD across childhood and adolescence were associated with adolescent hypomania [1] Significant, modest correlations between adolescent hypomanic and hyperactivity symptoms have also been reported [19, 20].

Given the existing knowledge gaps, we sought to characterize ADHD symptom trajectories across childhood and early adolescence from age 8 to 13, and to describe their prospective associated risk for subsequent hypomanic symptoms assessed between 21 and 23 years old. We also sought to distinguish inattention from hyperactivity to further examine the origins of the ADHD-BD overlap and investigate the prospective relationship between subtypes of ADHD (hyperactivity and inattentive symptoms) and hypomanic symptoms.

## Materials and methods

### Participants

The current study used data from the Avon Longitudinal Study of Parents and Children (ALSPAC), an ongoing longitudinal UK birth cohort study designed to investigate the factors associated with the development, health, and disease during childhood and beyond. [21–23] All women resident in Avon, UK, with expected dates of delivery between 1 April 1991 and 31 December 1992 were contacted and eligible for participation. [24] The study cohort consisted of 14,541 pregnancies and 13,988 children still alive at 1 year of age (see Supplement, for further details and Figure S1 for a flow chart detailing sample definition). Ethical approval was obtained from the ALSPAC Law and Ethics committee and the local research ethics committees. Informed consent was obtained from the parents of the children.

### Measures

#### ADHD across childhood and adolescence

ADHD at the age of 8, 10, and 13 was assessed using parental reports of the Development and Wellbeing Assessment (DAWBA). DAWBA is a validated instrument including both structured and semi-structured questions related to the International Classification of Diseases-10 (ICD-10) and Diagnostic and Statistical Manual of Mental Disorders fourth edition (DSM-IV) diagnostic criteria. [25] See Supplement for additional details on all measures. Items used for calculating the total scores for ADHD, and the total scores for its subtypes (inattention and hyperactivity) to obtain the trajectories can be found in Supplementary – Table 1. ADHD items prevalence in the cohort can be found in Supplementary – Table 2.

#### Clinically significant hypomanic symptoms in young people

Study participants completed the Hypomania Checklist Questionnaire (HCL-32), a self-report measure of lifetime experience of manic symptoms [26] comprising 32 items when they were 21–23 years old. Consistent with previous work, [27] we constructed a dichotomous clinically significant hypomanic symptoms variable; a) those with a symptom score of 14 or more (out of 32) were classed as having hypomania if they also reported b) at least one incident of “negative consequences” or of “negative plus positive consequences,” as a result of hypomanic symptoms, c) that mood changes caused a reaction in people close to the participant and d) that symptoms lasted for “4 days” or more. HCL-32 item prevalence in the cohort can be found in Supplementary – Table 3.

### Confounders

Child’s sex, and ethnicity were reported by the mother. Multiple adverse childhood experiences including but not limited to family psychopathology, socioeconomic status, and childhood abuse were assessed using the Family Adversity Index (FAI) during pregnancy and at 2 and 4 years (see Methods – Supplementary).

Borderline features were assessed using a face-to-face semi – structured interview, which was the Childhood Interview for DSM-IV Borderline Personality Disorder: UK Version (CI-BPD-UK), based on the borderline module of the Diagnostic Interview for DSM-IV Personality Disorders [28] at 11 years old (see further details in Methods – Supplement). We controlled for BPD traits as its highly associated with both ADHD and BD. [29–31]

### Statistical analysis

A multi-staged analysis plan was developed. In the first stage, we described the normative patterns of ADHD, subtypes of ADHD (hyperactivity only and inattention only), hypomanic symptoms and covariates across childhood, adolescence, and young adulthood using descriptive analysis implemented in SPSS, v29.

In the second stage as a primary analysis, we conducted latent class growth analyses (LCGA) using Mplus, v8 to potentially identify differing levels of ADHD symptoms across childhood and adolescence. We also conducted separate LCGAs for the subgroups of ADHD as a secondary analysis. The variables that were included in the LCGA analysis were DAWBA scores of ADHD at ages 8, 10, and 13 years. Several models were fitted by increasing the number of classes [32] from 2 to 6 classes. The best-fitting classification model was chosen using the following parameters: lower sample size–adjusted Bayesian information criteria, significant Vuong-Lo–Mendell–Rubin and Lo, Mendell, and Rubin likelihood ratio tests, higher entropy value, and the proportion of individuals in each class. [32] Missing values due to attrition were handled by the full information maximum likelihood estimation method. [33]

In the third stage, we conducted logistic regression analyses to explore the associations between ADHD trajectories and hypomania. Among 15,645 participants in the original sample of ALSPAC, 13,951 participants were lost to follow-up at age 21–23 years. Therefore, to deal with missingness which was unlikely to be missing at random, we conducted a weighted analysis using inverse probability to account for those lost to follow-up (See Supplementary – Methods). Characteristics associated with attrition at 21–23 years old were being a male, having a younger mother who had lower socioeconomic levels and having higher scores on FAI (see Supplementary – Table 4). Using the variables associated with selective dropout as the factors to predict missingness in our analysis sample, we fitted a logistic regression model (nonresponse vs response outcome) to determine weights for each individual using the inverse probability of response. The regression coefficients from this model were used to determine probability weights for the covariates in the primary and secondary analyses. Subsequently, unadjusted, and adjusted associations between ADHD (primary analyses) and subgroups of ADHD (secondary analyses) trajectories, and hypomanic symptoms in young adulthood were assessed using separate logistic regression analysis (i.e., three separate analyses). Additionally, we conducted sensitivity analyses to investigate whether reducing four HCL-32 items that are similar to ADHD items (i.e., talking fast, easily distracted, more energetic, and physically more active) would affect the results.

## Results

Table 1 shows the frequencies and descriptive values of the variables of interest in this study.

**Table 1.** Descriptive values of sociodemographic variables, ADHD symptom trajectories, and clinically significant hypomanic symptoms in ALSPAC Cohort^a^.

Variable	Mean (SD)	No. (%)
Child’s sex		
Female	-	7348 (8.9)
Male	-	7691 (51.1)
Ethnicity		
White	-	12062 (97.4)
Bangladeshi	-	7 (0.1)
Black African	-	11 (0.1)
Black Caribbean	-	76 (0.6)
Black Other	-	44 (0.4)
Chinese	-	30 (0.2)
Indian	-	54 (0.4)
Pakistani	-	22 (0.2)
Other	-	82 (0.7)
FAI, total scores^b^	4.38 (4.31)	-
BPD symptoms at 11 years, total score	0.35 (0.86)	-
DAWBA ADHD symptoms at 8 years total score (*n* = 7811), mean (SD)	4.21 (5.07)	-
DAWBA ADHD symptoms at 10 years total score (*n* = 7435), mean (SD)	3.87 (4.91)	-
DAWBA ADHD symptoms at 13 years total score (*n* = 6798), mean (SD)	3.42 (4.59)	-
Hypomanic symptoms total score at 21–23 years^c^	15.14 (16.00)	-
Clinically significant hypomanic symptoms at 21–23 years		
Yes	-	25 (1.8)
No	-	1348 (98.2)

*Note*: ADHD = Attention Deficit/hyperactivity disorder; BPD = Borderline Personality Disorder; DAWBA = Development and Well-Being Assessment; SD, Standard Deviation.

a Unweighted descriptive values for the total sample.

b The total Family Adversity Index scores for 3 time-points (i.e., during pregnancy, age 2 years, and age 4 years) were summed.

c Participants were asked to consider a time when they were in a “high or hyper” state and endorse a number of statements about their emotions, thoughts, and behaviours at that time.

### Primary analyses

#### Latent classes of ADHD

Table 2 shows the values of log-likelihood VLMR, ABIC, and number of other parameters for all models assessed. Overall, a 4-class model offered the best model fit and theoretical explanation (see Supplementary).

**Table 2.** BIC, VLMR likelihood test *p* values, and entropy for classes 2–6 of the DAWBA scores of ADHD, inattentive only, and hyperactivity only

Composite score of ADHD	AIC	BIC	ABIC	VLMR P-Value	LMRALT P-Value	Entropy
2 classes	121631.994	121689.284	121663.861	0.0000	0.0000	0.898
3 classes	118935.147	119013.921	118978.964	0.0000	0.0000	0.865
4 classes	117185.608	117285.865	117285.865	0.0000	0.0000	0.848
5 classes	116292.692	116414.433	116360.410	0.0549	0.0593	0.834
Hyperactivity only						
2 classes	92437.058	92494.411	92468.988	0.0000	0.0000	0.927
3 classes	89626.816	89705.678	89670.721	0.0000	0.0000	0.893
4 classes	87450.031	87550.400	87505.910	0.0038	0.0045	0.875
5 classes	85894.420	86016.296	85962.273	0.1924	0.1997	0.859
Inattentive only						
2 classes	100774.221	100831.546	100806.123	0.0000	0.0000	0.898
3 classes	98851.228	98930.049	98895.093	0.0000	0.0000	0.833
4 classes	95529.744	95630.062	95585.572	0.0000	0.0000	0.853
5 classes	94702.548	94824.362	94770.339	0.0061	0.0071	0.833

Abbreviations: ABIC = sample-size Adjusted Bayesian Information Criterion; AIC = Akaike Information Criterion; ADHD = Attention deficit/hyperactivity disorder; BIC = Bayesian information criterion; DAWBA = Development and Well-Being Assessment; VLMR = Vuong-Lo–Mendell–Rubin; LMRALT, Lo–Mendell–Rubin Adjusted LRT Test *p*-Value.

**Figure 1.**
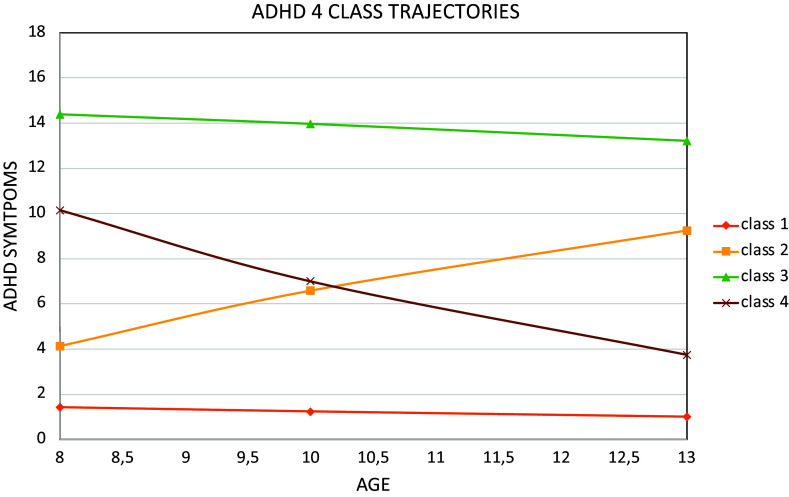
Four Class Model ADHD Symptoms – Developmental course of Development and Wellbeing Assessment (DAWBA) ADHD from 8 to 13 years old. The latent class growth analyses detected a best model fit for 4 classes. Class 1 (orange line on the bottom) represents individuals with persistent low levels of ADHD across time points. Class 2 (yellow line) represents individuals with increasing levels of ADHD. Class 3 (green line on the top) represents individuals with persistent high levels of ADHD. Class 4 (brown line) represents individuals with decreasing levels of inattentiveness.

Figure 1 shows the four trajectory classes: persistently low (66.1%, *N* = 6294), with ADHD symptoms that remained low at all time points; adolescence-increasing (10.3%, *N* = 981), with symptoms that began to increase later in adolescence; persistently high (9.1%, *N* = 865), with childhood onset ADHD symptoms that persisted into adolescence, with a very high probability of clinically significant ADHD symptoms at age 13; and remitting (14.5%, *N* = 1381), with clinically significant ADHD symptoms that began in childhood and remitted by adolescence.

#### ADHD classes and risk for clinically significant hypomanic symptoms

The weighted adjusted logistic regression model (with persistent low ADHD symptom levels as the reference) showed that persistently high levels of ADHD symptoms (OR = 2.36; CI 95% = 1.12–4.99; *p* = 0.024) and increasing levels of ADHD symptoms (OR = 3.60; CI 95% = 1.92–6.74; *p* < 0.001) were significantly associated with clinically significant hypomanic symptoms at the age of 21–23 compared to persistently low class (Table 3).

**Table 3.** Associations of latent classes of ADHD, hyperactivity only, and inattention only and risk of clinically significant hypomanic symptoms at 21–23 years^a^.

	Unadjusted Model			Adjusted Model		
	OR	95% CI	*P* Value	OR	95% CI	*P* Value
**ADHD composite score**						
Persistently low class (Ref)	-	-	<0.001			<0.001
Remitting class	0.00	0.00	0.992	0.00	0.00	0.990
Increasing class	3.88	2.12–7.08	<0.001	3.60	1.92–6.74	<0.001
Persistently high class	3.26	1.65–6.45	<0.001	2.36	1.12–4.99	0.024
Child’s sex	-	-	-	1.18	0.68–2.04	0.560
Child’s ethnicity	-	-	-	0.00	0.00	0.996
Family Adversity Index	-	-	-	1.19	1.13–1.25	<0.001
BPD traits at 11 years	-	-	-	1.26	1.07–1.49	0.007
**Hyperactivity only**						
Persistently low levels (Ref)	-	-	<0.001	-	-	0.005
Remitting class	0.00	0.00	0.992	0.00	0.00	0.991
Increasing class	2.03	0.91–4.53	0.084	0.92	0.35–2.43	0.868
Persistently high class	4.77	2.44–9.29	<0.001	3.47	1.69–7.12	<0.001
Child’s sex	-	-	-	0.97	0.55–1.71	0.911
Child’s ethnicity	-	-	-	0.00	0.00	0.996
Family Adversity Index	-	-	-	1.14	1.09–1.20	<0.001
BPD traits at 11 years	-	-	-	1.20	1.00–1.44	0.048
**Inattentive only**						
Persistently low levels (Ref)	-	-	0.011	-	-	0.039
Remitting class	0.00	0.00	0.995	0.00	0.00	0.995
Increasing class	3.58	1.47–8.70	0.005	3.25	1.32–8.04	0.011
Persistently high class	2.75	1.26–6.00	0.011	2.47	1.07–5.70	0.034
Child’s sex	-	-	-	0.51	0.24–1.09	0.082
Child’s ethnicity	-	-	-	0.00	0.00	0.997
Family Adversity Index	-	-	-	0.93	0.84–1.04	0.211
BPD traits at 11 years	-	-	-	1.37	1.10–1.70	0.004

*Note*: ADHD = Attention Deficit/hyperactivity disorder; BPD = Borderline Personality Disorder; DAWBA = Development and Well-Being Assessment; OR = Odds Ratio.

a All analyses were weighted for sex, ethnicity, maternal age, maternal socioeconomic status, preterm delivery, birthweight and family adversity; Adjusted Model: associations adjusted for child’s sex, child’s ethnicity, family adversity scores during pregnancy, at 2 years of age and 4 years of age, and BPD traits at 11 years.

#### Sensitivity analyses

Removal of hypomania items that were similar to ADHD items (four HCL-32 items; being more easily distracted, talking more, feeling more energetic and more active, and being physically more active) did not affect the results (Supplementary – Table 5).

### Secondary analyses

#### Latent classes of inattentive symptoms and risk for clinically significant hypomanic symptoms

Overall, a 4-class model offered the best fit and theoretical explanation (see Table 2, Supplementary, Table S6 and Table S7). Figure S2 shows the four trajectory classes: persistently low (63.8%, *N* = 6099); increasing (15.5%, *N* = 1486), persistently high (10.1%, *N* = 963), and remitting (6.5%, *N* = 1014).

The weighted adjusted logistic regression model (with persistent low levels as the reference) showed that persistently high levels class (OR = 2.47; CI 95% = 1.07–5.70; *p* = 0.034) and increasing levels class (OR = 3.25; CI 95% = 1.32–8.04; *p* = 0.011) were significantly associated with clinically significant hypomanic symptoms at age 21–23, compared to persistently low class (see Table 3).

#### Latent classes of hyperactivity symptoms and risk clinically significant for hypomanic symptoms

Overall, a 4-class model offered the best fit and theoretical explanation. We selected the 4-class model as this provided the best fit to the data and theoretical interpretation for hyperactivity (see Table 2 and Supplementary). Figure S3 shows the four trajectory classes: persistently low (74.3%, *N* = 7129); increasing (7.7%, *N* = 735), persistently high (7.5%, *N* = 722), and remitting (10.5%, *N* = 1011).

The weighted adjusted logistic regression model (with persistently low levels as the reference) showed that only persistently high levels class was significantly associated with clinically significant hypomanic symptoms at age 21–23 (OR = 3.47, CI 95% = 1.69–7.12, *p* < 0.001; Table 3) compared to persistently low class.

## Discussion

To the best of our knowledge, this is the first study to examine the extent to which, and how ADHD trajectories across childhood and adolescence, including ADHD subtypes, are associated with later clinically significant hypomanic symptoms. First, we identified a group of individuals characterized by persistently high, increasing, remitting, and persistently low levels of ADHD, inattentive, and hyperactivity symptoms across childhood and adolescence. Second, we found that persistently high levels and increasing levels of ADHD were independently associated with clinically significant hypomanic symptoms at the age of 21–23. Third, persistently high levels of hyperactivity and inattentive symptoms and increasing inattentive symptoms were also independently associated with subsequent clinically significant hypomanic symptoms.

Our results suggest that tracking ADHD symptoms over time in childhood and adolescence may help identify individuals at risk for clinically significant hypomanic symptoms. More specifically, our findings indicate that children and adolescents with persistently high ADHD symptoms (including hyperactivity and inattentive domains) and a greater cumulative burden of ADHD symptoms (including inattention domain only) are at higher risk of developing clinically significant hypomanic symptoms in young adulthood. The chronic levels of ADHD symptom trajectories may be reflecting children with ADHD with possibly developing BD since young people with ADHD plus BD compared to those with ADHD alone have a greater number of ADHD symptoms. [34] What we add to these findings is that even sub-threshold ADHD symptoms in childhood increasing in time could be a risk factor for developing clinically significant hypomanic symptoms later in life. Based on our results, chronicity and increasing levels could be critical to identify at-risk populations for BD and the dose-response signal adds validity to these findings.

When looking at the ADHD sub-domain classes, persistently high inattention and persistently high hyperactivity were significantly associated with clinically significant hypomanic symptoms. Interestingly, increasing inattentive levels were also significantly associated with clinically significant hypomanic symptoms but increasing hyperactivity levels were not. This is partly in line with previous research in which they found persistently high hyperactivity and inattention levels classes had the worst manic symptom severity scores. [35] Further, although they did not find a remitting class for inattentive levels, they did find hyperactivity symptom trajectories suggesting that hyperactivity symptoms wane more over time. They added that the remitting trajectory was associated with the highest rate of ADHD and the lowest rate of bipolar diagnoses. Building on these previous findings, our study adds that both ADHD sub-domains with the most favorable (persistently low) and remitting trajectory classes had the lowest risk for subsequent clinically significant hypomanic symptoms and both ADHD sub-domains wane over time. A pattern of inattention symptoms that are both chronically high and increasing over time appears to be particularly impactful in developing hypomanic symptoms.

There are many potential mechanisms by which ADHD symptoms may either lead to hypomanic symptoms or reflect comorbidity. While there are some common symptomatic features in both conditions, diagnostic criterion overlap may not entirely explain the comorbidity of both. [36] It has also been found that some shared clinical features are due to shared genetic factors. [37] For example, a twin study found that more than a quarter of the variance for hypomania was associated with shared genetic risk factors for ADHD traits and environmental influences appeared to have a negligible role in the associations between the two disorders. [1] Another large cohort study found that BD polygenic risk scores were strongly associated with childhood ADHD. [38] Additionally, a cross-disorder meta-analysis of the existing genome-wide association studies [39] (GWAS) provided evidence for genetic overlap between ADHD and BD such as G protein-coupled signalling already known for their role in hyperactivity and emotional behaviours. Further, another recent GWAS study [40] provided five novel risk loci showing concordant directions of effect for ADHD and BD. Future research is needed to clarify whether mechanisms driving associations between ADHD symptoms and hypomanic symptoms may differ depending on the pattern of ADHD symptoms that young people experience over time, including its subtypes. In line with previous research [1] our findings are unlikely to be explainable by symptom overlap given that exclusion of ADHD-like symptoms from the HCL variable did not modify our results.

There are several implications arising from the current findings. First, the present findings suggest that childhood and adolescent ADHD symptom trajectories, including its subtypes, may confer risk for clinically significant hypomanic symptoms in young adulthood. Practitioners and patients would be best served by completing multiple assessments of ADHD symptoms over time to identify individuals who are most at risk of future BD. Formally classifying child trajectories to target the reduction in high-risk trajectories and encourage preventative treatments is a critical next step. [41] This is crucial also because treatment earlier in the illness course is more effective. [42, 43] If replicated in individuals entering health service systems, the results can substantially help refine clinical staging models. [44] Future longitudinal research is needed to demonstrate the complex patterns of emergence of psychopathology in youth at risk for BD, along with their homotypic and heterotypic continuity, within a developmental framework utilizing multidisciplinary approaches. [45] Additionally, given the multidimensional nature of most mental disorders [46, 47], transdiagnosticity of these associations should also be examined. [48–51] This way, robust specific risk trajectories might also be identified. In the same vein, future research should also investigate the potential underlying mechanisms of the observed associations. Previous research has suggested that a history or current diagnosis of ADHD should be taken into account as a possible predictor of mixed or bipolar depression in patients with a major depressive episode (MDE). [52] For example, a study looking at the prevalence of ADHD in adult patients with BD observed a higher frequency of atypical depression (i.e., hypersomnia, hyperphagia, and increased appetite and weight gain) and a lower frequency of melancholic depression in the patients with the BD + ADHD group [53]. Another study found that mixed features during current MDE, earlier onset of depression before the age of 20, higher number of previous depressive and mood episodes, shorter duration of current MDE, and psychotic symptoms were more common in patients with comorbid major depression and ADHD comparing to the remaining sample [54]. Thus, one of the potential mechanisms future studies could investigate may be clinical characteristics suggestive of a bipolar depression diathesis (e.g., atypical depression features, psychotic symptoms, abrupt onset and offset, non-response to antidepressants, or antidepressant emergent elation, and family history of BD).

Our study has several limitations. First, despite our methods and results meeting several of the Bradford Hill criteria, [55] we have not demonstrated causation. Second, our cohort consisted of prepubertal children who tend to exhibit non-clinical symptomatology and derive from genetically heterogenous families. Therefore, our results may differ from young people who are seen in clinical settings. Third, ALSPAC has only one assessment timepoint of hypomanic symptoms. That is why, a baseline measure against which to compare stability symptoms over time was not available. Further, the HCL-32 was used as a measure of lifetime clinically significant hypomanic symptoms, but this will not always equate with a clinical diagnosis of hypomania and hypomanic symptoms were not clinically verified in the cohort. [56] The HCL-32 was self-reported, and this may have diminished the accuracy of the data due to recall biases. There is also a lack of chronology of hypomanic symptoms. Although we used a well-recognized cut-off score for lifetime hypomanic symptoms to improve the capacity of the HCL to identify clinical levels, amplified by measures of duration and impact on functioning, the combination of self-reports, parent reports and clinical structured interviews would be the ideal approach to increase the predictive value of our findings than the use of a single scale. [57] That is why, replicating these findings in help-seeking clinical populations utilizing both screening tools and clinical structured interviews is warranted. Fourth, only parent-reported data was available for all the ADHD assessments; however, symptoms may differ across settings and in interaction with different informants such as teachers and peers. [58] Fifth, we were only able to look at ADHD symptoms from age 8 to 13. However, the trajectory classes we have observed are in line with the highly dynamic changes in ADHD presentation from childhood to adulthood evident in the previous literature. [16, 59, 60] Sixth, the ALSPAC cohort was recruited in one region in Southwest England comprising mainly White participants, and therefore our findings may not generalize to other settings or birth cohorts. Additionally, although inverse probability weighting partially addressed cohort-specific patterns of non-response by adjusting samples to better represent the initial population, we cannot dismiss the biases that might stem from unmeasured factors that may influence missingness. [61] Seventh, although we focused on the associations with clinically significant hypomanic symptoms, given the multidimensional nature of most mental disorders, [44, 48, 50, 62] the same risk trajectories observed here may be associated with multiple types of disorders. Eight, there is the risk of residual confounding, as it is the case with all observational analyses. For example, childhood ADHD increases the risk of developing substance-related disorders [63–65] and there is a higher risk of developing BD in children and adolescents with ADHD with comorbid substance use disorders (SUDs). [66, 67] Past or current substance misuse may also confound the reliability of bipolar self-assessment screening [68] as SUDs can mimic affective episodes. [69] Cannabis use, particularly, may compound dopaminergic signalling in adolescence and lead to an increased propensity to experience hypomanic symptomatology. [70] Additionally, the psychostimulant methylphenidate, one of the most widely used medications for ADHD, may increase the risk of treatment-emergent mania in patients suffering from BD when it is used without a concomitant mood-stabilizing treatment. [71] However, it must also be noted that the available evidence with regards to manic switch risk with commonly used ADHD medications is limited and somewhat inconsistent. [72] For example, one study found that children with ADHD who were prescribed long-term methylphenidate (i.e., more than 365 days) had a lower risk of being diagnosed with BD. [73] Additionally, given the similar cognitive impairments in BD and ADHD, [72] if the observed associations between persistent and increasing inattention trajectories and clinically significant hypomanic symptoms are replicated in high-risk samples, identifying effective pro-cognitive treatments alongside mood stabilizers might be a helpful early intervention strategy for cognitive impairments. Lastly, although we were not able to control for ADHD medication use, evidence coming from other UK cohort studies highlights that the proportion of children with ADHD using medication remains lower than in North America, East Asia, France, and Central Europe [74, 75] possibly due to stigma and lack of recognition of the condition [76] and resource limitations. [77] Further, most of those who stop ADHD medication in adolescence do not have their prescriptions resumed in early adulthood. [78, 79] That is why, ADHD medication use might have had a negligible role in the associations we observed.

## Conclusions

We identified a pattern of chronically high ADHD and increasing ADHD symptoms across childhood and early adolescence as independent risk factors for clinically significant hypomanic symptoms in young adulthood compared to persistently low and decreasing levels. We have also found that a pattern of chronically high hyperactivity and inattention and increasing inattention levels were also independent risk factors for clinically significant hypomanic symptoms. These ADHD symptom profiles and trajectories represent a new and critical way of identifying at-risk populations for BD. Further validation in help-seeking clinical populations is warranted.

## Supporting information

Durdurak et al. supplementary material Durdurak et al. supplementary material

## Data Availability

ALSPAC data used within this study are accessible on request via an online proposal form. Please see http://www.bristol.ac.uk/alspac/researchers/access/ for further details. Please note that the ALSPAC website contains details of all data that are available through a fully searchable data dictionary and variable search tool (http://www.bristol.ac.uk/alspac/researchers/our-data/).

## Supplementary material

The supplementary material for this article can be found at http://doi.org/10.1192/j.eurpsy.2025.24.
