# Supplementary material for: ADHD symptom trajectories across childhood and early adolescence and risk for hypomanic symptoms in young adulthood
Source: Eur Psychiatry. 2025 Feb 19;68(1):e37. doi: 10.1192/j.eurpsy.2025.24 (PMC11883789; doi:10.1192/j.eurpsy.2025.24)
Supplement: Durdurak et al. supplementary material [file S0924933825000240sup001.docx]

**Supplementary Online Content**

**Methods.** Additional Information on Study Population, Measures, and Analyses

**Table 1.** The DAWBA ADHD Items

**Table 2.** DAWBA ADHD item prevalence in the cohort

**Table 3.** HCL-32 item prevalence in the cohort

**Table 4.** Differences in socio-demographic variables between non-participating and participating subjects in the study

**Table 5.** Associations of Latent Classes of ADHD and Risk of Hypomanic Symptoms with Reduced Items

**Table 6.** Classification Posterior Probabilities for 4-class model for Inattentiveness

**Table 7.** Classification Posterior Probabilities for 5-class model for Inattentiveness

**Figure 1. Attrition of participants from the ALSPAC cohort**

Figure 2. Four Class Model Inattentiveness

**Figure 3.** Four Class Model Hyperactivity Model

**References**

**Supplementary**

**Methods. Additional information on study population, measures, and analyses**

**Study Population**

The Avon Longitudinal Study of Parents and Children (ALSPAC) is a UK birth cohort study examining the determinants of development, health, and disease during childhood and beyond. ALSPAC recruited pregnant mothers in Avon whose anticipated delivery dates fell between April 1, 1991, and December 31, 1992. Initially, 14,541 pregnant women were enrolled in the study, and by July 19, 1999, they had either completed at least one questionnaire or attended a 'Children in Focus' clinic. Of these initial 14,541 pregnancies, there was a total of 14,676 foetuses. Among these, 14,477 were singletons, 195 were twins, 3 were triplets, and 1 was a quadruplet. There were 14,062 live births, and at the age of 1 year, 13,988 children were still alive. When the oldest children reached around 7 years old, an attempt was made to bolster the initial sample with eligible cases who had failed to join the study originally. Consequently, for variables collected from the age of 7 onwards, data is available for 14,701 children, representing an additional 713 children. Starting from the initial trimester of pregnancy, parents filled out postal questionnaires regarding the health and development of the study child. Additionally, the child participated in yearly assessment clinics, involving in-person interviews as well as psychological and physical evaluations. Study data were collected and managed using REDCap electronic data capture tools hosted at the University of Bristol ^1^ REDCap (Research Electronic Data Capture) is a secure, web-based software platform designed to support data capture for research studies. Data sources include self-report questionnaires, biological samples, clinical assessments and birth, medical, and educational records. The study website contains details of all the data available in a fully searchable format ([http://www.bris.ac.uk/alspac/researchers /data-access/data-dictionary/](http://www.bris.ac.uk/alspac/researchers%20/data-access/data-dictionary/)).

**Measures**

**Further details of the DAWBA:**

The DAWBA is a package of interviews, questionnaires and rating techniques designed to generate ICD-10 and DSM-IV or DSM-5 psychiatric diagnoses on 2-17 years old. A briefer questionnaire is administered to teacher. The interviews and questionnaires involve a mixture of closed questions such as "Does he ever worry?" and open-ended questions such as "Please describe in your own words what it is that he worries about?" and can either be administered by trained lay interviewers or else self-completed online. With the computer-administered interviews, the respondent types the open-ended answers into the text boxes. With the interviewer-administered interviews, it is the interviewer who transcribes the answers. The full DAWBA package covers the following diagnoses: Separation anxiety, Specific phobia, Social phobia, Panic disorder/agoraphobia, Post-traumatic stress disorder, Obsessive compulsive disorder, Generalized anxiety disorder, Body dysmorphic disorder, Disruptive mood dysregulation disorder, Major depression, ADHD/hyperkinesis, Oppositional defiant disorder, Conduct disorder, Eating disorders, including anorexia, bulimia and binge eating, Autism spectrum disorders, Tic disorders, including Tourette syndrome, and Bipolar Disorders. For each of these disorders, the interview asks about all the symptoms, and other criteria needed for an operationalized diagnosis according to both DSM-IV^2^ and the research diagnostic version of ICD-10 ^3^ The time frame of the interview is both the present and the recent past. Information from different informants is drawn together by a computer algorithm that predicts the likely diagnosis or diagnoses from responses to the closed questions, and generates six probability bands; < 0.1%, ~ 0.5%, ~ 3%, ~ 15%, ~ 50%, and > 70%. Each section contains around 20-25 questions, with skip-rules. For many disorders, the ICD-10 and DSM-IV diagnostic criteria stipulate that the symptoms need to have persisted for a specified period of time. That is why, the relevant section of the DAWBA interview focuses on the child’s symptoms over these stipulated periods as well. The DAWBA has considerable potential as an epidemiological measure and may prove to be of clinical value too ^4^

ADHD symptoms were measured using the “Attention and Activity” section of the DAWBA. Following previous work ^5^ symptoms were classed as present if mothers reported them occurring in their child “a little” or “a lot” more than in other children to create a count ranging from 0 to 18. First 9 hyperactivity items were used to calculate the total scores for hyperactivity only symptoms and the other 9 items were used to calculate the total scores for inattentive only subtype. To calculate the total scores for ADHD, all 18 items were used. See Table S1 for items used for calculating the total scores for ADHD and its subtypes.

**Hypomania Symptom Checklist (HCL-32):**

The HCL-32 is a self-reported questionnaire that assesses lifetime history of manic symptoms. It includes detailed assessments of bipolar mood, energy, and activity levels (in total 32 items). The instrument has been used extensively and validated in several studies ^6–9^ In clinical settings, it is considered to be a clinically useful screening instrument for hypomania and bipolar disorder type II.^10^ In ALSPAC, when the cohort were 21-22 years of age, postal and online questionnaires for assessing lifetime history of hypomanic symptoms, using HCL-32 were sent. The HCL-32 has been used as both a continuous and categorical measure of hypomanic symptoms ^11,12^ Respondents were asked to consider a time when they were in a “high or hyper” condition and endorse statements about their emotions, thoughts, and behaviour during this time. Although initially the HCL-32 was developed as a screening instrument for people with BD type II in people with recurrent depressive disorders, it is also a valid and sensitive tool for young, nonclinical populations ^13^. In our study, we used a categorical measure. Hypomanic symptoms were not clinically verified in the cohort.

**Confounders**

***Adverse Childhood Experiences using Family Adversity Index*:**

Family Adversity Index (FAI) total score was obtained using FAI index during pregnancy, and at 2 and 4 years, which includes items on (a)death or illness in family, (b) child’s experience of violent victimization(e.g., physical or sexual abuse), (c) inter‐parental conflict, (d) family disruption (e.g., mother got divorced or separated), (e) parental employment difficulties, (f) parental legal difficulties, (g) parental psychopathology (e.g., mother or father reported high levels of depression or anxiety symptomatology), (h) parental substance use(e.g., mother or father reported heavy alcohol consumption or illegal drug use), (i) financial hardship, and (j) housing inadequacies or instability.

***Borderline Personality Disorder Symptoms:***

Borderline Personality Disorder (BPD) was evaluated through a face-to-face semi-structured interview: the UK Childhood Interview for DSM-IV Borderline Personality Disorder (UK-CI-BPD; ^14^. This assessment tool is derived from the borderline module of the Diagnostic Interview for DSM-IV Personality Disorders (DIPD-IV; ^14^, a widely utilized semi-structured interview designed for assessing all DSM-IV Axis II disorders. The inter-rater and test–retest reliability of the DSM-III, DSM-III-R and DSM-IV versions of this measure have all proven to be good to excellent ^15^. It is the is first semi-structured interview assessing DSM-IV BPD in children and adolescents ^16,17^. The interview covers nine sections: intense inappropriate anger, affective instability, emptiness, identity disturbance, paranoid ideation/dissociation, frantic efforts to avoid abandonment, suicidal or self-mutilating behaviours, general impulsivity, and intense unstable relationships ^18^. If the symptom occurred daily or approximately 25% of the time, the symptom was classed as definitely present; and probable if it had occurred repeatedly but did not meet criterion for definitely present.

**Analyses**

**Determining the Optimal Number of Classes**

Model fit was assessed using the following parameters sample size–adjusted Bayesian information criterion, Lo-Mendell- Rubin likelihood ratio test, Vuong-Lo-Mendell-Rubin test, entropy value and proportion of individuals in each class. In line with previous studies utilising LCGA methodology, ABIC was given priority instead of Bayesian Information Criteria (BIC) because of previous studies indicating that it outperforms other information criteria indices such as Akaike Information Criteria (AIC) and Bayesian Information Criteria (BIC; ^19–22^, especially when small classes are present. Lower BIC values suggest better model fit. A significant VLMR value suggests that a k-class model fits the data better than a K − 1 class model. Entropy, a measure of the quality of classification, was additionally used to select the best model fit; an entropy value close to 1 is ideal. Finally, to decide the optimal class solution, an emphasis was also placed on proportion of individuals in each class, distinctiveness, and clinical relevance of the classes.

**Determining ADHD Classes**

We selected the 4-class model as this provided the best fit to the data and theoretical interpretation. It had the second lowest Sample-size Adjusted Bayesian Information Criteria (ABIC) value, and the Vuong-Lo-Mendell-Rubin test (VLMR) and Lo-Mendell-Rubin Adjusted LRT Test (LMRALT) P values suggested that it represented a significantly better fit than the 5-class model. While the 5-class model had a lower ABIC value, the entropy value was also lower compared to the 4-class model, suggesting a lower quality of classification. For these reasons, we selected the four-class solution over the five-class solution.

**Determining Inattentiveness Classes**

We also selected the 4-class model as this provided the best fit to the data and theoretical interpretation for inattentiveness. Although 5-class model had a lower ABIC value and VLMR and LMRALT tests were significant, the entropy value was lower compared to the 4-class model, suggesting a lower quality of classification. Additionally, although the smallest class in the five-class solution was not below the recommended 5%, 5-class model produced two very similar classes in terms of intercept and slope (which could be subsumed under the increasing levels of class). The difference between the four and five-class models was that the five-class solution added another increasing level class with a higher starting point as opposed to the other increasing levels class. The classification posterior probabilities for the five-class solution suggested it was difficult to distinguish those in higher levels increasing class to other classes, especially to those in the persistent high levels class. Classification posterior probabilities for the four and five class model can be seen in Table S9 and Table S10. For these reasons, we selected the four-class solution over the five-class solution.

**Determining Hyperactivity Classes**

We selected the 4-class model as this provided the best fit to the data and theoretical interpretation for hyperactivity. Very similar to the ADHD Classes output, 4-class model had the second lowest ABIC value, and the VLMR and LMRALT P values suggested that it represented a significantly better fit than the 5-class model. While the 5-class model had a lower ABIC value, the entropy value was higher compared to the 4-class model, suggesting a lower quality of classification. For these reasons, we selected the four-class solution over the five-class solution.

**Inverse Probability Weighting**

It is possible that selection may have induced bias due to non- random missingness of data in ALSPAC ^23^ To be able to assess the possibility of biased associations due to non-random missingness of the data, we used inverse probability weighting (IPW). This method has been recommended in epidemiological research over multiple imputation in situations where blocks of data are missing which is often the reason for attrition in ALSPAC where a variable is often missing due to non-participation in a clinic assessment visit ^23^ In IPW complete cases are weighted by the inverse of their probability of being a complete case and involves conducting an response/non-response model in order to account for any bias in patterns of association due to missingness ^24^.

Selective dropout was determined by comparing those participants who completed the HCL-32 questionnaire to those who dropped out, using logistic regression analyses. In line with previous publications based on this cohort ^25–27^ we used data from early time points in the ALSPAC dataset on child’s sex and ethnicity, mother’s socioeconomic status, maternal age, birthweight, preterm delivery and family adversity, to predict missingness in our analysis sample. The individuals associated with attrition at 21-23 years were more often males, and their mothers had higher scores on Family Adversity Index, they had lower socioeconomic status, had younger mother at birth (see Table S4).

Subsequently, we conducted IPW to account for those lost to follow-up. In accordance with previous research,^28,29^ we used the variables associated with selective drop-out as the independent variables and fitted a logistic regression model (response vs. nonresponse as outcome) to determine weights for each individual using the inverse probability of response. Associations were not similar for the unweighted and weighted data, and thus we used the weighted data in all subsequent analysis.

**Sensitivity Analyses**

In line with previous research^30^, to investigate whether removal of hypomania items from HCL-32 that are similar to ADHD items would affect the results, we removed the following 4 items from HCL-32;

- I am more easily distracted

- I talk more

- I feel more energetic and more active

- I am physically more active (sport etc.)

**Data Sources**

ALSPAC data used within this study are accessible on request via an online proposal form. Please see http://www.bristol.ac.uk/alspac/researchers/access/ for further details. Please note that the ALSPAC website contains details of all data that are available through a fully searchable data dictionary and variable search tool (http://www.bristol.ac.uk/alspac/researchers/our-data/).

**Table S1. The DAWBA ADHD Items**

|  |  |
| --- | --- |
| Hyperactivity Items |  |
|  | 1. Degree to which child often fidgeted in past 6 months relative to peers |
|  | 2. Degree to which child found it hard to sit down for long in past 6 months relative to peers |
|  | 3. Degree to which child ran or climbed about illicitly in past 6 months relative to peers |
|  | 4. Degree to which child found it hard to play quietly in past 6 months relative to peers |
|  | 5. Degree to which child found it hard to calm down in past 6 months relative to peers |
|  | 6. Degree to which child often blurted out answers in past 6 months relative to peers |
|  | 7. Degree to which child found it hard to wait own turn in past 6 months relative to peers |
|  | 8. Degree to which child often butted into conversations/games in past 6 months relative to peers |
|  | 9. Degree to which child often went on talking when asked to stop in past 6 months relative to peers |
| Inattentiveness Items |  |
|  | 1. Degree to which child often made careless mistakes in past 6 months relative to peers |
|  | 2. Degree to which child often lost interest in activities in past 6 months relative to peers |
|  | 3. Degree to which child often didn’t listen when addressed in past 6 months relative to peers |
|  | 4. Degree to which child often didn’t finish a job properly in past 6 months relative to peers |
|  | 5. Degree to which child often found it hard to get organised in past 6 months relative to peers |
|  | 6. Degree to which child often tried to get out of activities involving thought in past 6 months relative to peers |
|  | 7. Degree to which child often lost things needed for school in past 6 months relative to peers |
|  | 8. Degree to which child was easily distracted in past 6 months relative to peers |
|  | 9. Degree to which child was often forgetful in past 6 months relative to peers |

**Table S2. DAWBA ADHD item prevalence in the cohort**

|  | **8**  **(n=7811)**  **(%)** | **10**  **(n=7435)**  **(%)** | **13**  **(n=6798)**  **(%)** |
| --- | --- | --- | --- |
| **Hyperactivity Items** |  |  |  |
| Fidgeting | 2142 (13.7) | 1838 (11.7) | 1322 (8.4) |
| Hard to sit down | 1855 (11.9) | 1418 (9.1) | 910 (5.8) |
| Ran or climbed about illicitly | 1597 (10.2) | 776 (5.0) | 382 (2.4) |
| Find it hard to play quietly | 1353 (8.6) | 881 (5.6) | 615 (3.9) |
| Found it hard to calm down | 1630 (10.4) | 992 (6.3) | 657 (4.2) |
| Blurted our answers | 1479 (9.5) | 1316 (8.4) | 972 (6.2) |
| Hard to wait own turn | 1685 (10.8) | 1172 (7.5) | 786 (5.0) |
| Butted into conversations/games | 2520 (16.1) | 1911 (12.2) | 1365 (8.7) |
| Went on talking when asked to stop | 2754 (17.6) | 1980 (12.7) | 1548 (9.9) |
|  |  |  |  |
| **Inattentiveness Items** |  |  |  |
| Made careless mistakes | 2264 (14.5) | 2317 (14.8) | 1796 (11.5) |
| Lost interest in activities | 1507 (9.6) | 1428 (9.1) | 1155 (7.4) |
| Didn’t listen when addressed | 2396 (15.3) | 2100 (13.4) | 1820 (11.6) |
| Didn’t finish a job properly | 1888 (12.1) | 1963 (12.5) | 1790 (11.4) |
| Found it hard to get organised | 1820 (11.6) | 2058 (13.2) | 1893 (12.1) |
| Tried to get out of activities involving thought | 2020 (12.9) | 2365 (15.1) | 1971 (12.6) |
| Lost things needed for school | 1265 (8.1) | 1540 (9.8) | 1545 (9.9) |
| Easily distracted | 2420 (15.5) | 2262 (14.5) | 1951 (12.5) |
| Forgetful | 1717 (11.0) | 1887 (12.1) | 1719 (11.0) |

**Table S3. HCL-32 item prevalence in the cohort**

|  | **(n=1694)**  **(%)** |
| --- | --- |
| Needs less sleep | 1163 (7.4) |
| Feels more energetic and more active | 2810 (18.0) |
| More self-confident | 2694 (17.2) |
| Enjoys work more | 2616 (16.7) |
| More sociable | 2680 (17.1) |
| Wants to travel and/or do travel more | 2344 (15.0) |
| Drive faster or take more risks while driving | 491 (3.1) |
| Spends more/too much money | 1397 (8.9) |
| Takes more risks in daily life | 893 (5.7) |
| Physically more active | 2090 (13.4) |
| Plans more activities or projects | 2230 (14.3) |
| Has more ideas, is more creative | 2236 (14.3) |
| Less shy or inhibited | 2315 (14.8) |
| Wears more colourful and more extravagant clothes/make-up | 824 (5.3) |
| Wants to meet or actually do meet more people | 2005 (12.8) |
| More interested in sex and/or have increased sexual desire | 1826 (11.7) |
| More flirtatious and/or more sexually active | 1862 (11.9) |
| Talks more | 2704 (17.3) |
| Thinks faster | 1985 (12.7) |
| Makes more jokes or puns while talking | 2224 (14.2) |
| More easily distracted | 1155 (7.4) |
| Engages in lots of new things | 1527 (9.8) |
| Thoughts jump from topic to topic | 1284 (8.2) |
| Does things more quickly and more easily | 2033 (13.0) |
| More impatient and/or get irritable more easily | 559 (3.6) |
| Can be exhausting or irritating for others | 629 (4.0) |
| Gets into more quarrels | 226 (1.4) |
| Mood is higher, more optimistic | 2654 (17.0) |
| Drinks more coffee | 331 (2.1) |
| Smokes more cigarettes | 241 (1.5) |
| Drinks more alcohol | 571 (3.6) |
| Takes more drugs (both prescribed medications and recreational drugs) | 132 (0.8) |

**Table S4. Differences in socio-demographic variables between non-participating and participating subjects in the study for clinically hypomanic symptoms at age 21-23 years**

|  | **Non-participating group in the study** | | | **Participating group in the study** | | **Non-participating versus participating** | |
| --- | --- | --- | --- | --- | --- | --- | --- |
|  | *Mean* | *SD* | | *Mean* | *SD* | *OR (95% CI)* | *p* |
| Family Adversity Score | 4.54 4.39 | | | 3.46 3.67 | | 0.96 (0.94-0.98) <.001 | |
| Mother’s Socioeconomic Status | 52.22 13.53 | | | 56.45 13.45 | | 1.01 (1.00-1.01) 0.014 | |
| Birthweight | 3.38 58.59 | | | 3.42 5.39 | | 1.00 (1.00) 0.418 | |
| Maternal Age | 28.15 4.86 | | | 29.69 4.35 | | 1.04 (1.02-1.06) <.001 | |
|  | **Non-participating group in the study** | | | **Participating group in the study** | |  | |
|  | *N* | | *%* | *N* | *%* |  | |
| Sex |  | | |  | | 2.10 (1.79-2.46) <.001 | |
| Male/Female | 7082 / 6255 53.1 / 46.9 | | | 602 / 1091 35.6 / 64.6 | |  | |
| Ethnicity |  | | |  | | 0.95 (0.62-1.46) 0.815 | |
| White/Other | 10011 / 554 94.8 / 5.2 | | | 1506 / 58 96.3 / 3.7 | |  | |
| Preterm Delivery |  | | |  | | 0.76 (0.52-1.11) 0.158 | |
| Yes/No | 793 / 6663 10.6 / 89.4 | | | 946 / 63 93.8 / 6.2 | |  | |

**Table S5. Associations of Latent Classes of ADHD, Hyperactivity Only, and Inattention Only and Risk of Clinically Significant Hypomanic Symptoms at 21-23 Years with Reduced Items^a^**

|  | Unadjusted Model | | | Adjusted Model | | |
| --- | --- | --- | --- | --- | --- | --- |
|  | OR | 95% CI | *P* Value | OR | 95% CI | *P* Value |
| **Hypomanic Symptoms at 21-23 Years (with reduced items)** |  |  |  |  |  |  |
| **ADHD composite score** |  |  |  |  |  |  |
| ADHD persistently low levels class (Reference) | - | - | <0.001 | - | - | <0.001 |
| ADHD remitting class | 0.00 | 0.00 | 0.993 | 0.00 | 0.00 | 0.993 |
| ADHD increasing high class | 5.71 | 2.11-15.43 | 0.001 | 5.08 | 1.86-13.83 | 0.001 |
| ADHD persistently high class | 15.02 | 6.78-33.31 | <0.001 | 14.92 | 6.54-34.02 | <0.001 |
| Child’s sex | - | - | - | 1.52 | 0.74-3.11 | 0.252 |
| Child’s ethnicity | - | - | - | 0.00 | 0.00 | 0.996 |
| Family Adversity Index | - | - | - | 1.10 | 1.03-1.18 | 0.006 |
| BPD traits at 11 years | - | - | - | 1.18 | 0.99-1.41 | 0.072 |
| **Hyperactivity Only** |  |  |  |  |  |  |
| Persistently low levels (Ref) | - | - | <0.001 | - | - | <0.001 |
| Remitting class | 0.00 | 0.00 | 0.992 | 0.00 | 0.00 | 0.992 |
| Increasing class | 4.52 | 1.89-10.91 | <0.001 | 3.37 | 1.28-8.88 | 0.014 |
| Persistently high class | 10.63 | 5.03-22.47 | <0.001 | 8.10 | 4.02-20.12 | <0.001 |
| Child’s sex | - | - | - | 1.22 | 0.60-2.51 | 0.584 |
| Child’s ethnicity | - | - | - | 0.00 | 0.00 | 0.996 |
| Family Adversity Index | - | - | - | 1.07 | 1.01-1.14 | 0.034 |
| BPD traits at 11 years | - | - | - | 1.21 | 0.99-1.47 | 0.058 |
| **Inattentive Only** |  |  |  |  |  |  |
| Persistently low levels (Ref) | - | - | <0.001 | - | - | <0.001 |
| Remitting class | 0.00 | 0.00 | 0.993 | 0.00 | 0.00 | 0.993 |
| Increasing class | 5.71 | 2.11-15.43 | <0.001 | 5.08 | 1.86-13.83 | 0.001 |
| Persistently high class | 15.02 | 6.78-33.31 | <0.001 | 14.92 | 6.54-34.02 | <0.001 |
| Child’s sex | - | - | - | 1.52 | 0.74-3.11 | 0.252 |
| Child’s ethnicity | - | - | - | 0.00 | 0.00 | 0.996 |
| Family Adversity Index | - | - | - | 1.10 | 1.03-1.18 | 0.006 |
| BPD traits at 11 years | - | - | - | 1.18 | 0.99-1.41 | 0.072 |

Note: ADHD = Attention Deficit/hyperactivity disorder; BPD, Borderline Personality Disorder; DAWBA, Development and Well-Being Assessment; OR, Odds Ratio.

^a^All analyses were weighted for sex, ethnicity, maternal age, maternal socioeconomic status, preterm delivery, birthweight and family adversity; Adjusted Model: associations adjusted for BPD at 11 years, child’s sex, child’s ethnicity, and family adversity scores during pregnancy, at 2 years of age and 4 years of age.

**Table S6. Classification Posterior Probabilities for 4-class ADHD model**

|  | Increasing Levels Class | Decreasing Levels Class | Persistent Low Levels Class | Persistent High Levels Class |
| --- | --- | --- | --- | --- |
| Increasing Levels Class | 0.747 | 0.042 | 0.164 | 0.047 |
| Decreasing Levels Class | 0.033 | 0.791 | 0.122 | 0.054 |
| Persistent Low Levels Class | 0.011 | 0.009 | 0.980 | 0.000 |
| Persistent High Levels Class | 0.029 | 0.048 | 0.001 | 0.922 |

**Table S7. Classification Posterior Probabilities for 5-class model**

|  | Moderate Levels Increasing Class | Persistent High Levels Class | Decreasing Levels Class | Persistent Low Levels Class | High Levels Increasing Class |
| --- | --- | --- | --- | --- | --- |
| Moderate Levels Increasing Class | 0.663 | 0.011 | 0.035 | 0.241 | 0.051 |
| Persistent High Levels Class | 0.004 | 0.925 | 0.048 | 0.001 | 0.023 |
| Decreasing Levels Class | 0.029 | 0.050 | 0.816 | 0.094 | 0.010 |
| Persistent Low Levels Class | 0.012 | 0.000 | 0.010 | 0.977 | 0.001 |
| High Levels Increasing Class | 0.061 | 0.122 | 0.059 | 0.103 | 0.655 |

Figure S1. Attrition of participants from the ALSPAC cohort


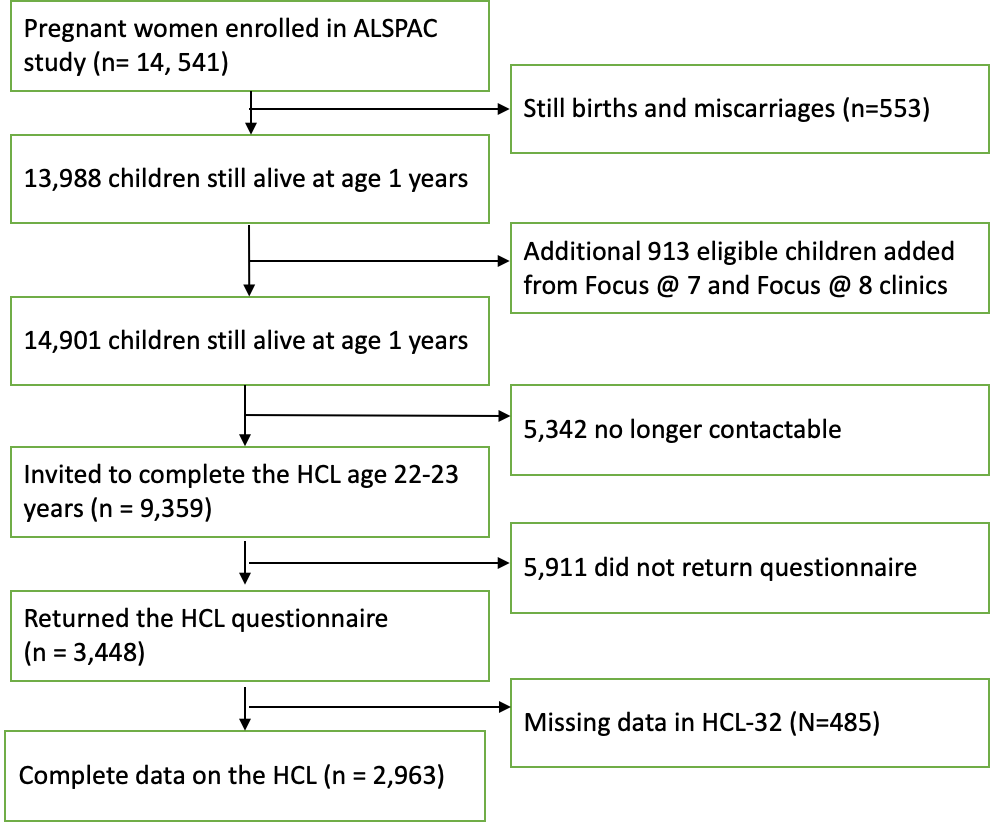


**Figure S2. Four Class Model Inattentive Symptoms**

Developmental course of Development and Wellbeing Assessment (DAWBA) inattentiveness subgroup from 8 to 13 years old. The latent class growth analyses detected a best model fit for 4 classes. Class 1 (orange line in the middle) represents individuals with increasing levels of inattentiveness across time points. Class 2 (yellow line) represents individuals with decreasing levels of inattentiveness. Class 3 (green line on the bottom) represents individuals with persistent low levels of inattentiveness. Class 4 (brown line) represents individuals with persistent high levels of inattentiveness.

**Figure S3. Four Class Model Hyperactivity Symptoms**

Developmental course of Development and Wellbeing Assessment (DAWBA) hyperactivity subgroup from 8 to 13 years old. The latent class growth analyses detected a best model fit for 4 classes. Class 1 (orange line on the top) represents individuals with persistent high levels of hyperactivity across time points. Class 2 (yellow line) represents individuals with decreasing levels of hyperactivity. Class 3 (green line on the bottom) represents individuals with persistent low levels of hyperactivity. Class 4 (brown line) represents individuals with increasing levels of hyperactivity.

**References**

1. Harris PA, Taylor R, Thielke R, Payne J, Gonzalez N, Conde JG. Research electronic data capture (REDCap)-A metadata-driven methodology and workflow process for providing translational research informatics support. *J Biomed Inform*. 2009;42(2):377-381. doi:10.1016/j.jbi.2008.08.010

2. American Psychiatric Association (APA). *Diagnostic and Statistical Manual of Mental Disorders.* Vol 29.; 1994. doi:10.3109/00048679509064964

3. World Health Organization. The ICD-10 classification of mental and behavioural disorders: diagnostic criteria for research. *World Health Organization*. 1993;2. doi:10.1007/978-3-319-24612-3_301942

4. Goodman R, Ford T, Richards H, Gatward R, Meltzer H. The Development and Well-Being Assessment: Description and initial validation of an integrated assessement of child and adolescent psychopathology. *J Child Psychol Psychiatry*. 2000;41(5):645-655. doi:10.1017/S0021963099005909

5. Powell V, Riglin L, Hammerton G, et al. What explains the link between childhood ADHD and adolescent depression? Investigating the role of peer relationships and academic attainment. *Eur Child Adolesc Psychiatry*. 2020;29(11):1581-1591. doi:10.1007/s00787-019-01463-w

6. Angst J, Azorin JM, Bowden CL, et al. Prevalence and characteristics of undiagnosed bipolar disorders in patients with a major depressive episode: The BRIDGE study. *Arch Gen Psychiatry*. 2011;68(8):791-799. doi:10.1001/archgenpsychiatry.2011.87

7. Forty L, Smith D, Jones L, et al. Identifying hypomanic features in major depressive disorder using the hypomania checklist (HCL-32). *J Affect Disord*. 2009;114(1-3):68-73. doi:10.1016/j.jad.2008.07.017

8. Meyer TD, Hammelstein P, Nilsson LG, Skeppar P, Adolfsson R, Angst J. The Hypomania Checklist (HCL-32): its factorial structure and association to indices of impairment in German and Swedish nonclinical samples. *Compr Psychiatry*. 2007;48(1):79-87. doi:10.1016/j.comppsych.2006.07.001

9. Meyer TD, Schrader J, Ridley M, Lex C. The Hypomania Checklist (HCL) - Systematic review of its properties to screen for bipolar disorders. *Compr Psychiatry*. 2014;55(5):1310-1321. doi:10.1016/j.comppsych.2014.03.002

10. Smith DJ, Anderson J, Zammit S, Meyer TD, Pell JP, Mackay D. Childhood IQ and risk of bipolar disorder in adulthood: prospective birth cohort study. *BJPsych Open*. 2015;1(1):74-80. doi:10.1192/bjpo.bp.115.000455

11. Court H, Forty L, Jones L, et al. Improving the psychometric utility of the hypomania checklist (HCL-32): A Rasch analysis approach. *J Affect Disord*. 2014;152-154(1):448-453. doi:10.1016/j.jad.2013.10.014

12. Carvalho AF, Takwoingi Y, Sales PMG, et al. Screening for bipolar spectrum disorders: A comprehensive meta-analysis of accuracy studies. *J Affect Disord*. 2015;172:337-346. doi:10.1016/j.jad.2014.10.024

13. Meyer TD, Schrader J, Ridley M, Lex C. The Hypomania Checklist (HCL) - Systematic review of its properties to screen for bipolar disorders. *Compr Psychiatry*. 2014;55(5):1310-1321. doi:10.1016/j.comppsych.2014.03.002

14. Zanarini MC. Zanarini Rating Scale for Borderline Personality Disorder (ZAN-BPD): A continuous measure of DSM-IV borderline psychopathology. *J Pers Disord*. 2003;17(3):233-242. doi:10.1521/pedi.17.3.233.22147

15. Zanarini MC, Frankenburg FR. Attainment and maintenance of reliability of axis I and II disorders over the course of a longitudinal study. *Compr Psychiatry*. 2001;42(5):369-374. doi:10.1053/comp.2001.24556

16. Winsper C, Hall J, Strauss VY, Wolke D. Aetiological pathways to Borderline Personality Disorder symptoms in early adolescence: Childhood dysregulated behaviour, maladaptive parenting and bully victimisation. *Borderline Personal Disord Emot Dysregul*. 2017;4(1):1-10. doi:10.1186/s40479-017-0060-x

17. Winsper C, Tang NKY, Marwaha S, et al. The sleep phenotype of Borderline Personality Disorder: A systematic review and meta-analysis. *Neurosci Biobehav Rev*. 2017;73:48-67. doi:10.1016/j.neubiorev.2016.12.008

18. Wolke D, Schreier A, Zanarini MC, Winsper C. Bullied by peers in childhood and borderline personality symptoms at 11 years of age: A prospective study. *J Child Psychol Psychiatry*. 2012;53(8):846-855. doi:10.1111/j.1469-7610.2012.02542.x

19. Jones HJ, Gage SH, Heron J, et al. Association of combined patterns of tobacco and cannabis use in adolescence with psychotic experiences. *JAMA Psychiatry*. 2018;75(3):240-246. doi:10.1001/jamapsychiatry.2017.4271

20. Morgan GB. Mixed Mode Latent Class Analysis: An Examination of Fit Index Performance for Classification. *Structural Equation Modeling*. 2015;22(1):76-86. doi:10.1080/10705511.2014.935751

21. Paksarian D, Cui L, Angst J, Ajdacic-Gross V, Rössler W, Merikangas KR. Latent trajectories of common mental health disorder risk across 3 decades of adulthood in a population-based cohort. *JAMA Psychiatry*. 2016;73(10):1023-1031. doi:10.1001/jamapsychiatry.2016.1921

22. Tofighi D, Enders CK. Identifying the correct number of classes in growth mixture models. *Advances in Latent Variable Mixture Models*. Published online 2008:317-341.

23. Weavers B, Heron J, Thapar AK, et al. The antecedents and outcomes of persistent and remitting adolescent depressive symptom trajectories: a longitudinal, population-based English study. *Lancet Psychiatry*. 2021;8(12):1053-1061. doi:10.1016/S2215-0366(21)00281-9

24. Seaman SR, White IR. Review of inverse probability weighting for dealing with missing data. *Stat Methods Med Res*. 2013;22(3):278-295. doi:10.1177/0962280210395740

25. Marwaha S, Winsper C, Bebbington P, Smith D. Cannabis use and hypomania in young people: A prospective analysis. *Schizophr Bull*. 2018;44(6):1267-1274. doi:10.1093/schbul/sbx158

26. Morales-Muñoz I, Palmer ER, Marwaha S, Mallikarjun PK, Upthegrove R. Persistent Childhood and Adolescent Anxiety and Risk for Psychosis: A Longitudinal Birth Cohort Study. *Biol Psychiatry*. 2022;92(4):275-282. doi:10.1016/j.biopsych.2021.12.003

27. Morales-Muñoz I, Mallikarjun PK, Chandan JS, Thayakaran R, Upthegrove R, Marwaha S. Impact of anxiety and depression across childhood and adolescence on adverse outcomes in young adulthood: a UK birth cohort study. *British Journal of Psychiatry*. 2023;45(4):212-220. doi:10.1192/bjp.2023.23

28. Morales-Muñoz I, Broome MR, Marwaha S. Association of Parent-Reported Sleep Problems in Early Childhood with Psychotic and Borderline Personality Disorder Symptoms in Adolescence. *JAMA Psychiatry*. 2020;77(12):1256-1265. doi:10.1001/jamapsychiatry.2020.1875

29. Morales-Muñoz I, Marwaha S, Upthegrove R, Cropley V. Role of Inflammation in Short Sleep Duration Across Childhood and Psychosis in Young Adulthood. *JAMA Psychiatry*. Published online 2024:1-9. doi:10.1001/jamapsychiatry.2024.0796

30. Hosang GM, Lichtenstein P, Ronald A, Lundström S, Taylor MJ. Association of Genetic and Environmental Risks for Attention-Deficit/Hyperactivity Disorder with Hypomanic Symptoms in Youths. *JAMA Psychiatry*. 2019;76(11):1150-1158. doi:10.1001/jamapsychiatry.2019.1949
